# Telehealth for global emergencies: Implications for coronavirus disease 2019 (COVID-19)

**DOI:** 10.1177/1357633X20916567

**Published:** 2020-03-20

**Authors:** Anthony C Smith, Emma Thomas, Centaine L Snoswell, Helen Haydon, Ateev Mehrotra, Jane Clemensen, Liam J Caffery

**Affiliations:** 1 Centre for Online Health, The University of Queensland, Australia; 2 Hans Christian Andersen Children’s Hospital, Denmark; 3 Centre for Innovative Medical Technology, University of Southern Denmark, Denmark; 4 Centre for Health Services Research, The University of Queensland, Australia; 5 Department of Health Care Policy, Harvard Medical School, USA

**Keywords:** Telehealth, telemedicine, pandemic, emergency, disaster management, sustainability, coronavirus, COVID-19

## Abstract

The current coronavirus (COVID-19) pandemic is again reminding us of the importance of using telehealth to deliver care, especially as means of reducing the risk of cross-contamination caused by close contact. For telehealth to be effective as part of an emergency response it first needs to become a routinely used part of our health system. Hence, it is time to step back and ask why telehealth is not mainstreamed. In this article, we highlight key requirements for this to occur. Strategies to ensure that telehealth is used regularly in acute, post-acute and emergency situations, alongside conventional service delivery methods, include flexible funding arrangements, training and accrediting our health workforce. Telehealth uptake also requires a significant change in management effort and the redesign of existing models of care. Implementing telehealth proactively rather than reactively is more likely to generate greater benefits in the long-term, and help with the everyday (and emergency) challenges in healthcare.

## Introduction

The number of cases of coronavirus disease 2019 (COVID-19) is increasing rapidly and, as of 11 March 2020, the World Health Organization has declared that this can be characterised as a pandemic.^1^ Governments are preparing for the worst, quickly realising the impact that COVID-19 is having on health services and the global economy. Amidst the avalanche of reports concerning the spread of the virus, there is also recognition (again) that telehealth ‘could’ play a critical role in the global response.

Of course, telehealth is ideal for the management of communicable diseases. A key factor in slowing the transmission of a virus is ‘social distancing’^2^ thus decreasing person-to-person contact. For patients with COVID-19, or those concerned that they might be infected, telehealth can help with remote assessment (triage) and the provision of care. For people not infected with the COVID-19 virus, especially those at higher risk of being affected (e.g. older adults with pre-existing medical conditions), telehealth can provide convenient access to routine care without the risk of exposure in a congested hospital or in medical practice waiting rooms.

However, for telehealth to be effective during the current COVID-19 pandemic and future events, we must ensure that telehealth is appropriately integrated into our health service, and treated as a ‘business as usual’ modality. The aim of this article is to outline key requirements to ensure that the value of telehealth is fully realised, not only in emergencies (such as pandemics) but also in everyday practice.

## Previous use of telehealth in emergency situations

Telehealth has a number of key strengths that can enhance an emergency response when environmental or biological hazards present. During infectious disease outbreaks, telehealth can enable remote triaging of care and provide rapidly accessible information through technology – such as chatbots, as seen in Singapore during COVID-19.^3^ Telehealth can also assist with disease diagnosis via video consultations with health professionals. Various applications exist for providing ongoing care as demonstrated by a hospital in the USA where physicians are currently using telehealth to care for COVID-19 patients remotely.^4^ Additionally, telehealth can enable people to navigate the health system and access routine care during an infectious disease outbreak.

The current COVID-19 event is not the first time that government agencies and healthcare providers have turned to telehealth in response to disaster situations. The North Atlantic Treaty Alliance (NATO) (an intergovernmental military alliance between 29 members including North American and European countries), developed a Multinational Telemedicine System in 2000 that has been deployed with their military forces during various crises.^5^ Through solutions such as person-deployable portable telemedicine kits and satellite linkage, areas in need have received health support from medical experts located in other countries.^6^ During hurricanes Harvey and Irma,^7^ private telemedicine companies provided care to victims relocated from their homes and primary care providers. Following the Severe Acute Respiratory Syndrome (SARS) pandemic in 2003, China began exploring telehealth and integrated electronic medical systems for use in similar situations in the future.^8^ During severe prolonged droughts in Australia, the health department introduced new funding through the Medicare Benefits Schedule (MBS) to allow clinicians to provide additional mental health services via videoconferencing.^9^ In 2019, similar mental health services were also offered to people affected by the bushfires.^9^

Whilst the potential benefits of telehealth are clear,^10–12^ the uptake of telehealth in emergency situations has been limited. As an example, the funding provided by the Australian government to support the delivery of online (videoconference) mental health services to people affected in the bushfire crisis seems to have had little impact.^13^ Despite the availability of MBS funding, claims data shows only four telehealth visits were provided in the first three months.

## Barriers to the use of telehealth and strategies to address them

Outside of emergency situations, the overall uptake of telehealth has been slow and fragmented.^14,15^ Substantial efforts have gone into scaling-up the routine use of telehealth, often with limited success. In Australia, despite the introduction of generous financial incentives for specialist videoconsultations, telehealth represented less than 1% of all specialist consultations provided.^16^ The experience in the USA has been similar, where less than 1% of people living in rural areas have ever experienced telehealth. Reasons for the low uptake of telehealth are multifaceted and diverse, but factors such as clinician willingness, financial reimbursement and (re)organisation of the health system may be to blame.

### Clinician willingness and acceptance of telehealth

The limited uptake of telehealth services is mostly attributable to clinician’s unwillingness to adopt telehealth.^17^ A timely telehealth response to emergencies such as the COVID-19 outbreak, calls for a health workforce that is skilled and capable of switching delivery modes, as required. Relying just on sporadic uptake of telehealth, as in times of emergency, is problematic.

Why the unwillingness to adopt telehealth? Telehealth is disruptive,^18^ complex^19^ and requires clinicians to learn new methods of consulting.^20^ Clinician acceptance of telehealth relies on them perceiving telehealth as effective, safe and normal.^17^ Clinicians may not be knowledgeable and aware of telehealth, ^21^ which is not surprising given there is limited telehealth training in medical, nursing and allied health pre-registration curricula.^22^

Regular telehealth practice leads to more sustainable models of care,^23^ and a telehealth-ready workforce. Ensuring the health workforce is telehealth-ready will require telehealth to be included in training and education.^24^ Therefore, it is imperative to include telehealth in curricula and to mandate post-graduate telehealth accreditation. This will send a clear message to current and future healthcare professionals that telehealth is a legitimate part of usual care. Furthermore it may increase readiness to use telehealth in every day practice, and in times of emergency.

### Reimbursement

Appropriate remuneration is needed for all telehealth services. Traditionally, the lack of funding has been blamed for the slow uptake of telehealth.^25^ Constraints for funding associated with geographical location and service type have also limited expansion of telehealth in city locations. For example, in Australia, funding is predominantly focused on medical consultations delivered by videoconference for patients in rural and remote locations. This is problematic because telehealth is just as useful for people living in metropolitan locations. In the case of COVID-19, city locations are most at risk because of greater population density.^26^ In other emergencies, certain communities may be affected and therefore require increased access to specialist health services, hence the importance of telehealth capability, irrespective of rurality.

Temporary funding methods may be an appropriate way of dealing with ad-hoc emergencies such as COVID-19. Depending on the nature of the emergency, prioritising telehealth funding for specific services or for a selected patient group (such as older people with a respiratory illness) could help address high-risk and high-demand situations. This funding could be authorised by the government at short notice and decommissioned after the emergency.

Countries have begun to address the reimbursement barriers associated with COVID-19. In March 2020, emergency supplemental funding legislation for coronavirus was passed in the USA which allows the federal government to expand telehealth to patients in metropolitan areas and also allows physicians to care for patients in their homes.^27^ In Australia, there have been similar calls to relax restrictions on general-practitioner-provided telehealth consultations.^28^ Whilst remuneration for telehealth services is an important requirement, a focus on funding alone will not generate an effective telehealth service. Other critical factors need to be considered.

### Organisation of the healthcare system

Dependency on individual clinicians to lead telehealth is not a sustainable approach to the expansion of telehealth. Telehealth adoption requires a whole-system strategy. Embedding telehealth into routine service delivery, by all healthcare providers, is the most effective way of ensuring telehealth can be readily used during emergencies. This requires operational telehealth networks, telehealth policies and procedures, and technology infrastructure that can be scaled-up during times of disaster. Telehealth is a disruptive process, so there is a need for effective change-management strategies to support clinicians with limited telehealth experience. Furthermore, simulated testing of telehealth applications for emergency situations is also a useful way of ensuring that workflow processes are clear and effective.^29^

Multiple resources are available to support disaster preparedness and response strategies. For example, the American Telemedicine Association Emergency and Response special interest group has developed a framework and infrastructure checklist that could be used at local, regional and national levels during disaster events.^30^ The NATO Multinational Telemedicine System (described above) resulted in the development of a system, supported by guidelines and technology solutions, which is able to interconnect various national telemedicine capabilities for use during disasters.^5^

In the absence of any formal telehealth strategy, it is important to make telehealth guidelines available to assist with co-ordination and delivery of telehealth services during an emergency event. This information needs to suit all stakeholders, including patients, clinicians, health service providers and funders.^31^ International health agencies such as the World Health Organization, national centres for disease control, and health departments have been disseminating real-time information about COVID-19 via their websites and social media outlets^32,33^ and have a very important role to play in advocating for the use of telehealth via these channels. These organisations can increase awareness of telehealth, provide specific recommendations on effective telehealth use, and validate the importance of telehealth’s role in the healthcare sector.

## Conclusion

While we may not be able to accurately predict the timing of natural disasters and infectious pandemics, we can be sure that they will present again in the future. The COVID-19 experience is not a first, and nor will it be the last. Telehealth does have a critical role in emergency responses. Advantages of telehealth include the ability to: rapidly deploy large numbers of providers; facilitate triage so that front-line providers are not overwhelmed with new presentations; supply clinical services when local clinics or hospitals are damaged or unable to meet demand; and decrease the risk of communicable diseases which are transmitted by person-to-person contact.

There are also limitations to the use of telehealth. Some consultations require physical examinations that may be difficult to perform remotely (e.g. auscultation) and diagnostics (e.g. imaging, cultures) which cannot be done remotely. It is important that clinician training highlights the limitations of telehealth and informs of alternative methods of information gathering that can be used in these situation. These situations also highlight the importance of providing care via telehealth to non-infected people during an infectious pandemic. This can reduce contamination when it is necessary to see an infected patient in-person

It is important that the development of a telehealth strategy to deal with global and national emergency responses is built on the premise that telehealth becomes a mainstream component of our health system. The question is, ‘How can this be realised?’ The answer is quite straightforward…

- Ensure that all health professionals receive appropriate education and training;
- Introduce telehealth accreditation for health professionals;
- Provide funding which adequately covers the cost of providing telehealth;
- Redesign clinical models of care;
- Support all stakeholders with an effective communication and change management strategy;
- Establish systems to manage telehealth services on a routine basis.

With these important requirements in place, the consideration of whether telehealth could be used in emergencies will become redundant as it should just happen.
